# Wild Mushrooms as a Source of Bioactive Compounds and Their Antioxidant Properties—Preliminary Studies

**DOI:** 10.3390/foods13162612

**Published:** 2024-08-20

**Authors:** Izabela Bolesławska, Ilona Górna, Marta Sobota, Natasza Bolesławska-Król, Juliusz Przysławski, Marcin Szymański

**Affiliations:** 1 Department of Bromatology, Poznan University of Medical Sciences, 3 Rokietnicka Street, 60-806 Poznan, Poland; ibolesla@ump.edu.pl (I.B.); martasobota13@wp.pl (M.S.); jprzysla@ump.edu.pl (J.P.); 2 Student Society of Radiotherapy, Collegium Medicum, University of Zielona Gora, 28 Zyty Street, 65-046 Zielona Góra, Poland; natmeff@gmail.com; 3 Centre for Advanced Technologies, Adam Mickiewicz University of Poznan, 10 University of Poznan Street, 61-614 Poznan, Poland; marcin.szymanski@amu.edu.pl

**Keywords:** wild mushrooms, bioactive ingredients, polyphenols, antioxidant properties, heavy metals

## Abstract

The aim of this study was to preliminarily determine the content of bioactive components in the fruiting bodies of four previously unstudied mushroom species: *Aleuria aurantia*, *Phallus hadriani*, *Phanus conchatus*, *Geastrum pectinatum*, their antioxidant activity and the content of polyphenols, minerals and heavy metals. Methods: Determination of active compounds by gas chromatography-mass spectrometry was carried out in addition to thermogravimetric determinations, quantitative determination of total polyphenols by spectrophotometry using Folin-Ciocalteu reagent, determination of antioxidant activity using 2,2-diphenyl-1-picryl hydrazyl radical (DPPH) and 2,2′-azino-di-[3-ethylbentiazoline sulphonated] (ATBS). In addition, spectrometric analysis of selected minerals and heavy metals was performed by inductively coupled plasma optical emission spectroscopy (ICP-OES). Results: The mushrooms analysed varied in terms of their bioactive constituents. They contained components with varying effects on human health, including fatty acids, oleamide, 1,2-dipalmitoylglycerol, (2-phenyl-1,3-dioxolan-4-yl)-methyl ester of oleic acid, deoxyspergualin, 2-methylenocholestan-3-ol, hexadecanoamide, isoallochan, 2,6-diaminopurine, and adenine. All contained polyphenols and varying amounts of minerals (calcium, magnesium, iron, zinc, potassium, phosphorus, sodium, copper, silicon and manganese) and exhibited antioxidant properties of varying potency. No exceedances of the permissible concentration of lead and cadmium were observed in any of them. Conclusions: All of the mushrooms studied can provide material for the extraction of various bioactive compounds with physiological effects. In addition, the presence of polyphenols and minerals, as well as antioxidant properties and the absence of exceeding the permissible concentration of heavy metals, indicate that these species could be interesting material in the design of foods with health-promoting properties, nutraceuticals or dietary supplements. However, the use of the fruiting bodies of these mushrooms requires mandatory toxicological and clinical studies.

## 1. Introduction

Global food problems, particularly pronounced in developing countries as a result of rapid population growth, limited land resources, climate change, wars, natural disasters and catastrophes [1,2], are forcing the search for low-cost sources of food and/or the enrichment of traditional foods with value-enhancing ingredients. The consequence is a growing need to search for new sources of food and/or ingredients that can enrich it. In addition, consumers are increasingly aware of the relationship between food and their health [3].

An excellent complement to conventional dietary foods is mushrooms and their ingredients, which can be used to design and manufacture new food products, functional foods or nutraceuticals. Mushrooms are high in carbohydrates, including fibre, a source of vitamins (especially B vitamins), ergosterol—a precursor of vitamin D—and minerals. Their amino acid composition, comparable to animal proteins, allows them to compete with products of animal origin (meat, eggs, milk) [4,5]. In addition, they contain bioactive components with medicinal properties that can be used to enrich food [6,7].

The use of mushrooms and their preparations in medicine and pharmacy has been of unflagging interest for a long time. Many therapeutic effects induced by mushrooms or bioactive compounds derived from mushrooms have been reported, including antihyperlipidemic [8,9], antidiabetic [10,11], antiallergic [12], antimicrobial [13], hepatoprotective [14], anti-inflammatory [15], antioxidant [8,16], antiviral [17], cytotoxic [13,18], and immunomodulatory [19] by stimulating the expression of genes encoding proteins involved in the immune response, analgesic, antidepressant [20,21], neuroprotective [16] and nephroprotective effects [22]. Some mushrooms have also been shown to have an effect on the prevention and supportive treatment of various forms of cancer [23]. Bioactive components of mushrooms of the genera Agaricus, Aurcularia, Trametes, Pleurotos, and Ganoderma have been approved as adjuvants in traditional anticancer, anti-infective, antidiabetic or anti-atherosclerotic therapies [24]. Novel enzymes belonging to ribotoxin-like proteins have been isolated in wild fungi. These enzymes have been shown to have multiple activities (e.g., antiviral, antifungal, endonuclease, cytotoxicity), thus finding potential applications in biomedicine as a diagnostic and therapeutic agent. These ribotoxin-like proteins have been isolated and well characterised in many wild fungi (e.g., Ageritin from *Cyclocybe aegerita* [25], Ostreatin from *Pleurotus ostreatus* [26], Edulitins from *Boletus edulis* [27] and Gambositin from *Calocybe gambosa* [28], Eryngitins from *Pleurotus eryngii* [29]). Interestingly, some mushrooms that show medicinal activity are considered inedible or even poisonous, such as Amanita muscaria [30]. Despite this, one of its compounds, muscimol and its analogues, has been proposed for treating some neurodegenerative diseases or cerebral ischemia due to its neuroprotective effects [31]. More recent studies have shown it to have significant cytotoxic [32], analgesic [33], antioxidant and anti-inflammatory effects [34]. 

Many nutraceuticals, a wide range of supplements and cosmeceuticals based on bioactive ingredients derived from carpophores, mycelium or spore powder are already available on the market [35], and exploratory clinical trials of the efficacy of mushroom micro-pharmaceuticals are in the early stages [36].

Recently, there has also been a marked increase in interest in the potential use of fungi in the food sector. One of the leading directions is the use of medicinal mushroom preparations to develop functional foods. This encourages the search for beneficial pharmacological properties in as yet unstudied wild mushrooms and their application in medicine, pharmacy and food technology. Identifying the composition and action, cultivation and commercialisation of new types of mushrooms that can act as a food ingredient, nutraceutical, or drug is a promising research area [7]. Even more so given global factors such as food, health and social care, and environmental shortages. The ability of fungi to grow rapidly on undemanding or unconventional substrates is an added advantage [4].

*Aleuria aurantia*, *Phallus hadriani*, *Phanus conchatus*, and *Geastrum pectinatum* are fungi growing wild in forested areas in Central Europe, but they have not been previously studied for their composition and bioactive constituents, with the exception of the antioxidant status and heavy metal content of *Geastrum pectinatum* [37]. *Aleuria aurantia* (Pers.) Fuckel (family: *Pyronemataceae*) is a common fungus found not only in Europe but worldwide [38,39]. It is an edible, tasteless saprotroph, very often used to decorate dishes. *Panus conchatus* (Bull.) Fr. (family: *Polyporaceae)* is a saprotroph, an edible but indigestible fungus [40], found in Mexico and Peru in addition to Europe [41]. In contrast, *Geastrum pectinatum* (Pers.) (family: *Geastraceae*) and *Phallus hadriani* (Vent.) (family: *Phallaceae*) are rare species, considered inedible [42,43]. *Phallus hadriani* in egg form shows no toxic properties and is considered a delicacy by the French. This species shows a repulsive odour when ‘hatched’ in its mature form, which may suggest that these fungi are toxic or inedible [44]. *Geastrum pectinatum*, on the other hand, is inedible due to its fibrousness and hard digestibility but is not poisonous; dried, it can make an attractive table decoration [45]. *Geastrum pectinatum* is found in mixed and coniferous forests, while *Phallus hadriani* grows on sandy and dry coastal dunes on the seacoast and is less common inland [46]. Both are cosmopolitan, occurring on all continents except Antarctica [47,48].

The use of fungi as an ingredient in food or dietary supplements is challenging. For many fungal species, the toxicological properties are not fully recognised [49]. Misidentification of inedible or poisonous mushrooms as edible can cause poisoning [50]. Mushrooms may also contain toxic metals, pesticides, process contaminants, radioactive isotopes, microbial contaminants, inherited toxins from the environment, processing, degradation and spoilage, and food-contact materials [51]. Some can cause allergies and severe infections in humans, albeit with low frequency [52]. Therefore, the growing interest in mycotherapy requires a solid commitment to expanding research and proposing ‘safe’ products.

The aim of our study was to preliminarily identify bioactive constituents in the fruiting bodies of four mushroom species that have not been studied so far: *Aleuria aurantia*, *Phallus hadriani*, *Phanus conchatus*, and *Geastrum pectinatum* with a view to their use in future dietary supplements or functional foods. In addition, an evaluation of thermal stability, determination of the content of active compounds, determination of antioxidant activity, determination of the content of selected macro- and micronutrients, and determination of heavy metal and aluminium contamination of these fungal species were carried out.

## 2. Materials and Methods

### 2.1. Research Material

The study’s material consisted of fruiting bodies of four fungal species: *Aleuria aurantia*, *Phallus hadriani*, *Phanus conchatus*, and *Geastrum pectinatum*, collected at full development stage. For the present study, 10 fruiting bodies of each genus were collected from different forest sites in the Wielkopolska region of Poland in the temperate climate zone from an area not exceeding 500 m^2^ for a particular species. Fungal fruiting bodies were collected from different forest sites in the temperate climate zone of the Wielkopolska region of Poland. The collected mushrooms were examined and identified taxonomically. Preliminary identification was made on the basis of macroscopic features based on published descriptions and textbooks [39,46,53,54]. Morphological features such as size, colouration, texture shape and edge of the fruit were taken into account, as well as other characteristic features (odour, pith size, stem length, gill spacing and attachment). Identification of fungal nomenclature was confirmed using Index Fungorum [55] and Mycobank [56].

Depending on the species, the raw material was freeze-dried under vacuum using the Heto PowerDry PL3000 Freeze Dryer (Thermo Fisher Scientific, Waltham, MA, USA) at −50 °C or air dried at room temperature (27 °C) for 10 days. This was dictated by the low water content of the *Geastrum pectinatum* fruiting body and the risk of spores being aspirated from the tender fruiting body when the freeze dryer was turned off. The prepared fruiting bodies were ground into powder using an electric grinder and packed in airtight bags, where they were stored under ambient conditions (27 °C) until analysis. Samples for the study collected in 2017–2019 were prepared and stored for each species separately. They were deposited and analysed in the analytical laboratory at the Centre for Advanced Technology, Adam Mickiewicz University, Poznań. A full description of the samples is presented in Table 1.

### 2.2. Characteristics of the Fungal Species Studied

*Aleuria aurantia* (Pers.) Fuckel (family: *Pyronemataceae*), in the classification according to Index Fungorum [55]: *Aleuria*, *Pyronemataceae*, *Pezizales*, *Pezizomycetidae*, *Pezizomycetes*, *Pezizomycotina*, *Ascomycota*, Fungi. The discovery of this taxon was first made by mycologist Christiaan Hendrik Persoon in 1800, and he named it *Peziza aurantia*. The present name, recognised by Index Fungorum, was given to it in 1870 by Karl Wilhelm Gottlieb Leopold Fuckel and transferred to the genus *Aleuria* [41]. The fruiting bodies of *Aleuria aurantia* appear from late summer to autumn and occur mostly in groups and in great numbers in sunny and open places on the ground. It prefers sandy and clay soils and is very often one of the first organisms to emerge on massively deposited sand and soil heaps after flooding. It strongly avoids calcareous soils [57]. The diameter of the fruiting body of *Aleuria aurantia* ranges from 2 to 10 cm. In young specimens, it is almost spherical and then bowl-shaped with curved edges, while in older specimens, it is flat, round, lobed, and curved with wavy edges. *Aleuria aurantia* does not have a stem. The inner genital layer, where the spores are formed, is smooth. It has a colouration ranging from yellow-red through orange to tin-brown-red. The outer side of this taxon is delicately velvety, taking on a whitish to pale yellowish-ochre colouration [58]. The flesh is very thin, crumbly, watery, and grey-white in colour, with a very poor taste and smell, but it smells very pleasant when crushed. Spores—are colourless, elliptically oblong and covered with reticulate ornamentation. Their size ranges from 16–24 × 8–12 µm [46].

*Panus conchatus* (Bull.) Fr. (family: *Polyporaceae*), classification entry according to Index Fungorum [55]: *Panus*, *Polyporaceae*, *Polyporales*, *Incertae*, *sedes*, *Agaricomycetes*, *Agaricomycotina*, *Basidiomycota*, Fungi. It was first discovered by Bulliard in 1787 and named *Agaricus conchatus*. In 1838, Fries transferred it to the genus *Panus*, which is now recognised by the Index Fungorum. *Panus conchatus* is in the sparse group of the lamellate hymenophores. In contrast, the vast majority of species in this order have a tubular hymenophore. However, phylogenetic studies have shown that it is closely related to the sailfish family [59]. The cap of the lichen is 4–12 cm wide, shell-shaped or lingual, with a funnel-shaped depression in the middle. The surface is smooth or velvety, and the colour is ochre to reddish brown in older specimens and lilac in young specimens. The lamellae are pale lilac to ochre in colour and deeply converging on the stem. The height of the stem is 1–3 cm. The thickness oscillates between 1 cm and 2 cm, it is non-centred with a felt-like surface in an ochre brown to lilac colour, and the flesh is leathery, phloem-like and white. The spores are white, elliptical, non-amyloid with a size of 7–5 × 2.5–3 µm Its fruiting bodies grow from summer to autumn on the dead wood of deciduous trees, mainly on stumps and trunks [48]. It is most common in moist riparian forests, mostly in clusters. It grows on a variety of tree species, most commonly on oaks, birches, poplars and aspens. The fruiting bodies emerge from July to November [49]. It is recorded in North, Central and South America, Europe and Asia. It is very widely distributed; however, it is rare everywhere [60].

*Geastrum pectinatum* (Pers.) (family: *Geastraceae*) position in the classification according to Index Fungorum [55]: *Geastrum Geastraceae*, *Geastrales*, *Phallomycetidae*, *Agaricomycetes*, *Agaricomycotina*, *Basidiomycota*, Fungi. Young fruiting bodies are spherical and closed with a diameter of 1–4 cm. Slightly convex at the top and covered with a thin layer of mycelium. The outer covering breaks almost halfway into 4–9 sharp and spreading arms. The star-spangled fruiting bodies reach a diameter of 3–9 cm. The arms are thick, and in young fruiting bodies, they are covered with a mealy and grey-brown coating. In older specimens, the arms tilt downwards, and the shoulders are then darker and usually cracked. The inner cover is initially globular, then flattened and dark brown. The apophysis is distinct, broad and radially wrinkled. The mouth at the apex is conical, brown, and crested with 18–25 notches, which are surrounded by a roll. The spores are papillary and globular [54]. It appears from April to October in clusters of several or occurs alone. 

**Figure 1:**
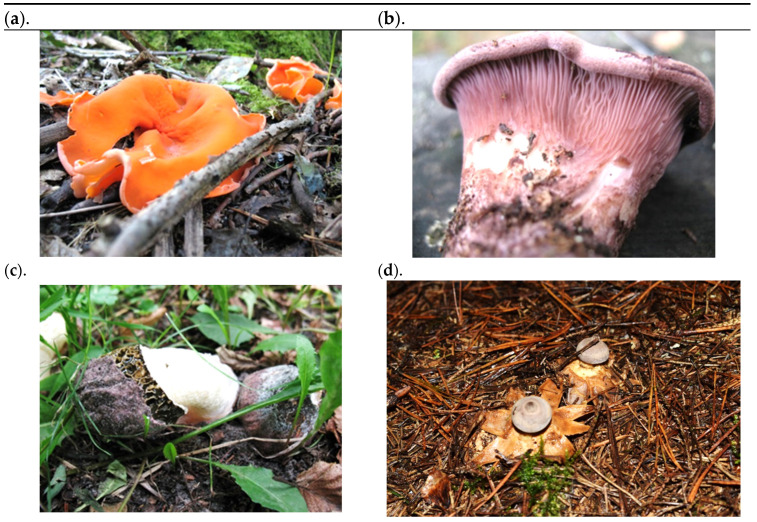
Photographs of the fungal species analysed: (**a**) *Aleuria aurantia*, (**b**) *Panus conchatus*, (**c**) *Phallus hadriani*, (**d**) *Geastrum pectinatum* (photographs and collection Marcin Szymański).

*Phallus hadriani* (Vent.) (family: *Phallaceae*) classification heading according to Index Fungorum [55]: *Phallus*, *Phallaceae*, *Phallales*, *Phallomycetidae*, *Agaricomycetes*, *Agaricomycotina*, *Basidiomycota*, Fungi. Young fruiting bodies occur as an ovoid or spherical egg sheathed in a hard brown membrane. The receptacle is composed of a shaft up to 20 cm long, usually 6–10 cm long, and a vagina with a whitish, reticulate head. The colouration of the receptacle is greyish, pinkish or purplish. The flesh of the shaft is spongy, whitish, and has numerous chambers, and it is very friable. Pileus with algal mass, ≤30 mm high × 25 mm in diameter, cylindrical, tapering at the top, hollow. Fruiting bodies occur in summer and autumn. Spores are 3–5 × 1–2 μm, ellipsoidal, glabrous, and smooth [46,47]. The fruiting bodies we examined occurred in egg form. Photographs of individual fungi are shown in Figure 1.

### 2.3. Identification of Active Compounds in Ethanolic Extracts of Analysed Fungi

#### 2.3.1. Extraction of Bioactive Compounds

In a 50-mL round-bottom flask, 1000 g of powdered mushroom fruiting body was weighed and poured into 10 mL of absolute ethanol (Merck, Kenilworth, NJ, USA), then heated on a heating bowl at boiling point for 10 min. After cooling, it was filtered and concentrated to about 5 mL in a stream of air. The extract thus prepared was filtered using a 0.45 µm syringe filter.

#### 2.3.2. Analysis of Extracts by GC-MS

Ethanolic extracts of whole fungal samples were analysed for the presence of various compounds by GC-MS technique [61]. The analysis was carried out in the analytical laboratory at the Centre for Advanced Technologies, Adam Mickiewicz University, Poznań. GC-MS determined the composition of the ethanolic extracts on a Bruker gas chromatograph with mass detection. Separation conditions: electron energy: 70 eV, ion source at 200 °C, column: silica VF-5 ms (30 m × 0.25 mm × 0.39), df = 0.25 µm, carrier gas: helium, flow rates: 1 mL/min. Analysis time: 55 min.

One microlitre of the sample was injected in pulse mode without spitting onto a fused silica column. Identification time was based on the retention time of the volatile and semi-volatile components in the column. The relative percentage of each extract component was expressed as a percentage with peak area normalisation [62].

The relationships analysed were different in each species. Identification of the components was performed by comparing their retention rates and mass spectral fragmentation patterns with compounds stored in the National Institute for Standard and Technology (NIST) Chemical Library Version 2.4 [63]. Peak area measurement and data processing were performed using Plot Chromatograms and Spectra software (MS Work station 8).

### 2.4. Determination of Total Polyphenols with Folin-Ciocalteu Reagent

Determination of total polyphenols in extracts from four selected mushroom species was carried out by a spectrophotometric method using the Folin-Ciocalteu reagent from Merck and a 20% sodium carbonate (Na_2_CO_3_) solution from POCh [64]. Polyphenols in plant extracts react with specific redox reagents (FC reagent) to form a blue complex, which can be quantified by visible-light spectrophotometry [65]. After adding the FC reagent and Na_2_CO_3_ solution and obtaining coloured solutions, their absorbance maxima were read at λ = 760 nm on a spectrophotometer Lambda 35 (Perkin—Elmer). The content of phenolic compounds was calculated from a calibration curve made using gallic acid as a standard. The gallic acid curve was constructed from six dilutions of gallic acid solution. Determinations were performed in five replicates. Results were expressed as the converted polyphenol content as mg GAE (gallic acid)/100 g of dried product.

#### 2.4.1. Determination of Standard Solutions of Gallic Acid

A stock solution of gallic acid was prepared by adding distilled water to 10.0 mg of gallic acid to a volume of 50 mL. The resulting concentration of gallic acid was 0.2 mg/mL. Further, solutions of 0.01, 0.02, 0.03, 0.04, 0.05, and 0.06 mg of the standard compound in the sample were prepared in 6 aluminium foil-wrapped measuring tubes. Then 0.5 mL of FC was added to each tube, followed after 1 min by 2 mL of 20% sodium carbonate. The tubes were made up to 10 mL with water, and after 30 min, the absorbance was measured at λ = 760 nm against the reference sample, which was a mixture of the above reagents, omitting the gallic acid solution.

#### 2.4.2. Determination of Total Polyphenols in the Test Extract

Weighed 2.500 g of raw material was extracted with 50 mL of water in a boiling water bath under a reflux condenser for 30 min. The extracts were then filtered through cotton wool and made up to 50 mL with distilled water to obtain a stock solution. Subsequently, 5 mL of water, 0.1 mL of the stock solution, 0.5 mL of Folin-Ciocalteu reagent and, after two minutes, 2 mL of 20% sodium carbonate were added to 6 aluminium foil-wrapped measuring tubes and made up to 10 mL with water. Absorbance was measured after 30 min, at λ = 760 nm.

### 2.5. Determination of Antioxidant Activity

#### 2.5.1. Determination of Antioxidant Activity by the DPPH Method, Determination of the IC_50_ Parameter

The antioxidant activity of methanolic and aqueous plant extracts was assessed according to the modified method of Brand-Wiliams et al. [66] using the synthetic radical DPPH (1,1-diphenyl-2-picrylhydrazyl) from MERCK. The antioxidant properties were spectrophotometrically evaluated by measuring the absorbance of the tested extracts at λ = 515 nm on a spectrophotometer Lambda 35 (Perkin—Elmer). Based on the results obtained, the IC50 parameter (effective concentration) was determined, indicating the concentration of the extract required to neutralise 50% of the free radicals in the reaction medium [67].

One hour before the assay series was performed, a solution of 0.1 mM DPPH in methanol was prepared. A series of dilutions (100 mg/mL) were made from the previously prepared stock solution. The dilutions were determined on the fly so that the plot, Aa = aCpr + b (where Aa-antioxidant activity of a solution of a given concentration, Cpr is the concentration of the sample, and a, b are the parameters of the straight line), was linear over the range tested. 0.2 mL of each concentration was taken into a further five vials protected with aluminium foil, and then 3.9 mL of methanolic DPPH radical solution was added. Absorbance was measured after 30 min at λ = 515 nm, against a reference (mixture of 0.2 mL methanol/water and 3.9 mL DPPH radical).

The ability to reduce the DPPH radical was calculated based on the formula:

Aa = (A0 − Ai/A0) × 100%

where: Aa is the antioxidant activity [%], Ai is the mean absorbance of the test solution, A0 is the mean absorbance of the DPPH radical.

The IC_50_ parameter for the extracts tested was calculated by transforming the equation of the straight line Aa = aCpr + b relative to Cpr and after substitution Aa = 50.

#### 2.5.2. Determination of Antioxidant Activity Using ABTS

This method measures the ability of antioxidants to neutralise the stable radical cation of 2,2′-azinobis(3-ethylbenzthiazoline-6-sulphonic acid) (ABTS˙+) [68]. The decrease in colour intensity is proportional to the antioxidant content of the test sample. The antioxidant activity using the ABTS reagent, expressed by the IC_50_ parameter, was tested spectrophotometrically on a spectrophotometer Lambda 35 (Perkin–Elmer) by measuring the absorbance of extracts from the tested mushroom fruiting bodies at λ = 734 nm.

The reduction capacity of the free ABTS cation radicals by the tested extracts was calculated using the formula:

X = (Ap/AABTS˙+) × 100 (%)

where: X is the percentage of residual ABTS, Ap is the mean absorbance of the test sample, and AABTS˙+ is the mean absorbance of the solution of ABTS˙+ cation radicals (without antioxidant added).

ABTS+ solution was prepared by dissolving 0, 19,204 g ABTS f (Merck) in a previously prepared 2.45 mM potassium persulphate solution (Chempur). A 7.0 mM solution of ABTS˙+ cation radicals was obtained. The solution was then incubated in a dark place at room temperature for 24 h. After this time, the solution was diluted so that its absorbance, measured at λ = 734 nm, was approximately 0.85. Five replicates were then made for each dilution of the stock extract. ABTS was added and mixed, and then the samples were sealed in light-protected containers. A spectrophotometric measurement was made at λ = 734 nm 10 min after the addition of ABTS. The reference sample was water. The absorbance of the ABTS solution (50 µL of MILLIPORE deionised water and 1.0 mL of ABTS solution) was measured before the absorbance measurement. To perform the assay, 50 µL of the extract of the given dilution and 1, 0 mL of the A solution were added sequentially to 2 mL Eppendorf type tubes.

### 2.6. Determination of Selected Elements by ICP-OES Method

#### 2.6.1. Mineralisation of Raw Materials

Raw material balances were mineralised in 10 mL of concentrated Ultranal nitric acid (Merck) on a CEM Corporation USA Mars 5 microwave mineraliser and then supplemented with deionised water (Millipore) to a volume of 15 mL. Samples prepared in this way were percolated and then subjected to ICP-OES analysis. The mineralisation parameters are shown in Table 2.

#### 2.6.2. Determination of Elements by ICP-OES Method

The elements in the fruiting bodies of the four mushroom species studied were determined using a Varian ICP-OES emission spectrophotometer. For the analysis of each element in the fruiting bodies of the tested mushrooms, the determination was repeated three times in 2 parallel weighings. The content of individual elements was calculated using the equation of the standard curve for each component: y = a x + b. ICP standards purchased from FLUCA-ANALYTICAL were used to determine the standard curves of the analysed elements. Based on the signal intensity of the sample obtained by mineralising an appropriate weight of raw material in nitric acid, the content of mg of the element in 1 L of the solution was calculated. Taking into account a total sample volume of 15 mL, the amount of mg of the element in 15 mL of solution was obtained, and after taking into account the sample weights, the element content in 1 g of dry matter was obtained. A calibration curve was drawn up for each element, and from this, the concentrations of the elements concerned were determined.

### 2.7. Thermogravimetric Evaluation of Mushroom Fruiting Bodies

Thermogravimetric analysis is used to study phase transformations using an instrument called a thermowell. The thermowell allows the mass of the sample to be recorded continuously during the heating process. The results of the TG analysis are obtained in the form of temperature dependence curves of the sample mass. Such curves can be used, among other things, to determine the most suitable temperatures for thermal and/or industrial food processing. The method is particularly suitable for analysing products from the pharmaceutical and food industries [69].

**Figure 2:**
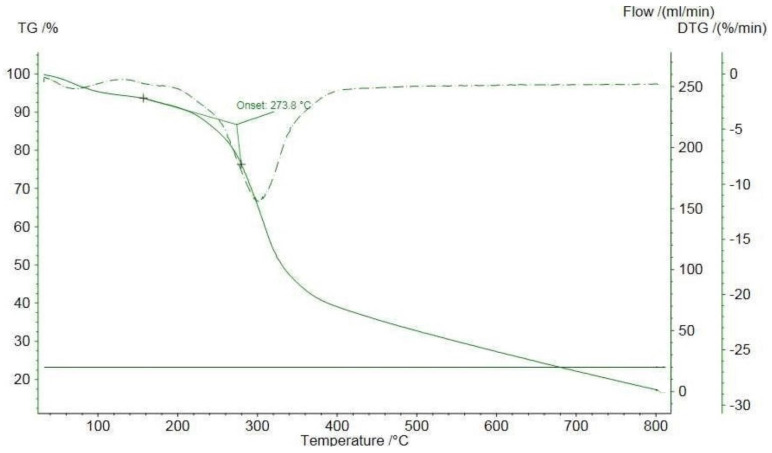
Thermogravimetric analysis of PH sample, created with NETZSCH proteus software by Marcin Szymański. The solid line denotes the TG curve (TG is a function: G = f(T), where: T—temperature)—the change in mass of the sample as it is heated or cooled, the dashed line denotes the DTG curve (DTG is the derivative of dG/dt = F(T); where: T = β∙t (β—heating rate of the sample; t—time)—the change in decomposition rate of the substance with increasing temperature. As a result of the TG analysis, a curve is obtained on which the y-axis is located the change in mass of the sample (decreasing downwards), and on the x-axis is time or temperature. On the TG curve, it is possible to observe the steps associated with the loss or gain of mass of the sample during heating or cooling. In parallel, a differential thermogravimetric (DTG) analysis is performed. The first derivative of the thermogravimetric curve against time (t) or temperature (T) is then obtained. The DTG curve represents the change in decomposition rate of a substance with an increase or decrease in temperature. In contrast, the total mass loss of the sample is equal to the area of the peak on this curve.

Thermogravimetric analysis was carried out to determine the thermal stability of the tested mushroom fruiting bodies using a NETZSCH TG 209 F1 Libra apparatus at 30–800 °C, in a nitrogen atmosphere, with a nitrogen flow of 20 mL/min, in Al_2_O_3_ crucibles. Samples were pyrolysed at elevated temperatures. The temperature of the first mass changes of the test material and the temperature of the thermal decomposition of the sample were recorded. The temperature until the thermal decomposition of the sample, where the weight loss is negligible, indicates the thermal stability of the product. An example of the course of the analysis is shown in Figure 2.

### 2.8. Statistical Analysis

Identification of active compounds in ethanolic extracts of the fungi analysed was performed in triplicate. Data collection was performed using Brucker—Plot Chromatograms and Spectra software, and data analysis was performed using NIST MS Search 2.2 Library 2014. For total polyphenol content, results are presented as mean, standard deviation, and coefficient of variation v%. Quantitative data analysis was performed using the Microsoft Excel 2016 software package. The statistical analysis used the method of least squares, which involves fitting a straight line to all measured points in such a way that the sum of the squares of the differences between the actual and estimated values is as small as possible. Selected elements by ICP-OES were analysed using Origin 9.0 software (OriginLab Corporation, Northampton, MA, USA). Concentrations were expressed as mean ± standard deviation. Thermogravimetric evaluation of fungal fruiting bodies was carried out in triplicate for each fungus species. The _IC50_ determination for antioxidant activity using DPPH and ABTS was repeated five times for each extract. Results are presented as mean ± standard deviation.

## 3. Results

### 3.1. Identification of Active Compounds in the Ethanol Extracts of the Analysed Fungi

In the ethanol extract of *Geastrum pectinatum*, the highest percentage content of 2-methylene-cholestan-3-ol was found (50.23%). The percentage of oleamide content was 20.05%. Ethyl isoallocholate (11.61%), hexadecanoamide (8.394%), and 18,19-secoyohimban-19-oicacid,16,17,20,21-tetradehydro-16-(hydroxymethyl)-, methyl ester (1.218%) were present in smaller amounts.

The ethanolic extract of *Aleuria aurantia* had the highest percentage of linoleic acid content (77.72%). Palmitic acid (11.90%), oleic acid (5.26%), 2-methylene-cholesteran-3-ol (2.65%), and D-mannose (1.28%) were present in smaller percentages. 

**Figure 3:**
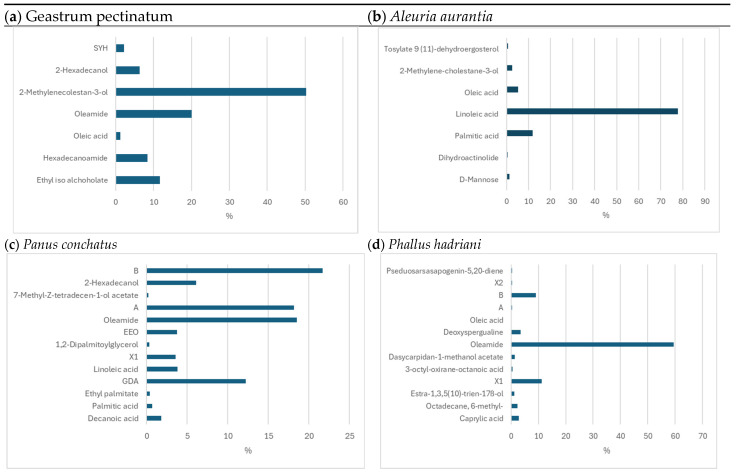
Identification of active compounds in extracts from (**a**) *Geastrum pectinatum:* Ethyl iso allocholate, hexadecanoamide, oleic acid, oleamide, 2-methylenocholestan-3-ol, 2-hexadecanol, SYH—(18,19-secoyohimban-19-oic acid, and 16,17,20,21-tetradehydro-16-(hydroxymethyl)-, methyl ester, (15ß,16E)-), (**b**) *Aleuria aurantia*: D-mannose, dihydroactinolide, palmitic acid, linoleic acid, oleic acid, 2-methylenocholestan-3-ol, 9 (11)-dehydroergosterol tosylate, (**c**) *Panus conchatus:* decanoic acid, palmitic acid, ethyl palmitate, GDA—gibbane-1,10-dicarboxylic acid, 2,3-epoxy- 4a,7-dihydroxy-1-methyl-8-methylene-, 1,4a-lactone, 10-methyl ester, (1a,2ß,3ß,4aa,4ba,10ß)-), linoleic acid, X1—not identified, 1,2-dipalmitoylglycerol, EEO—3-ethyl-5-(2-ethylbutyl)—Octadecane, oleamide, A—17′-acetoxy-3′ß-methyl- (5′ß) Spiro (1,3-dithion)-2,2′-(androstan-3′-ol), 7-methyl-Z-tetradecen-1-ol acetate, 2-hexadecanol, B—1,3,5,7-tetramethyl-2,4-Diselena-6,8-dioxatricyclo[3. 3.1.1(3,7)]decane (**d**) *Phallus hadriani;* caproic acid, octadecane, 6-methyl-, ester-1,3,5(10)-trien-17ß-ol, 3-octyl-oxirane-octanoic acid, dasycarpidan-1-methanol acetate, oleamide, deoxyspergualine, oleic acid, X1, X2—not identified, A—(Engl. 8,14-Seco-3,19-epoxyandrostane-8,14-dione, 17-acetoxy-3ß-methoxy-4,4-dimethyl-), B—(2-phenyl-1,3-dioxolan-4-yl)-methyl ester of oleic acid), pseduosarsasapogenin-5,20-diene.

*Panus conchatus* extract had the highest percentage content for 1,3,5,7-tetramethyl-2,4-diselena-6,8-dioxatricyclo[3.3.1. 1(3,7)]decane) (21.714%) and oleamide (18.491%), 17′-acetoxy-3′ß-methyl-(5′ß) spiro (1,3-dithioate)-2,2′-(androstan-3′-ol) (18.147%), 2-hexadecanol 6.093%, 3-ethyl-5-(2-ethylbutyl)-octadecane (3.749%), and linoleic acid (3.81%). The ethanolic extract of *Phallus hadriani* showed the highest percentage of oleamide (59.413%). In lower percentages, the following were present: (2-phenyl-1,3-dioxolan-4-yl)-methyl ester of oleic acid (9.117%), deoxyspergualin (3.52%), caproic acid (2.803%), 6-methyl octadecane (2.403%), estra-1,3,5(10)-trien-17ß-ol (1.219%), and dasycarpidan-1-methanol acetate (1.322%). The percentage of each compound in the samples analysed is shown in Figure 3.

### 3.2. Total Polyphenols Content

*Phallus hadriani* extract had the highest percentage polyphenol content of 1.501 ± 0.025 mg GAE/100 g s.m of the samples analysed. A slightly lower content of these compounds was present in the extract from *Aleuria aurantia* and *Phanus conchatus* (0.906 ± 0.025 and 0.900 ± 0.024 mg GAE/100 g s.m, respectively). The lowest polyphenol content was found in the extract of *Geastrum pectinatum* (0.617 ± 0.016 mg GAE/100 g s.m). The results of the percentage content of polyphenols per gallic acid in the samples tested are presented in Table 3.

### 3.3. Determination of Antioxidant Activity by the Method with DPPH

Aqueous extracts of *Phallus hadriani* (IC_50_ 4.74 mg/mL) had the strongest activity against free radicals. The aqueous extract of *Aleuria aurantia*: (IC_50_ 17.06 mg/mL), the methanolic extract of *Phallus hadriani* (IC_50_ 18.19 mg/mL), the aqueous extract of *Panus conchatus* (IC_50_ 21.26 mg/mL) were less active. The aqueous extract of *Geastrum pectinatum* (IC_50_ 37.92 mg/mL), the methanolic extract of *Panus conchatus* (IC_50_ 35.21 mg/mL), and *Aleuria aurantia* (IC_50_ 40.86 mg/mL) showed average oxidative activity. The methanolic extract of *Geastrum pectinatum* (IC_50_ 84.13 mg/mL) showed the weakest antioxidant activity. The results are summarised in Table 4.

### 3.4. Determination of Antioxidant Activity by the Method with ABTS

The aqueous extract from *Panus hadriani* had the strongest activity against free radicals with an IC_50_ of 10.21 mg/mL. The methanolic extract of *Phallus hadriani* (IC_50_ 31.71 mg/mL), the aqueous extract of *Panus conchatus* (IC_50_ 47.16 mg/mL) and aqueous extract of *Aleuria aurantia* (IC_50_ 53.43 mg/mL) showed a slightly weaker effect. The aqueous extract of *Geastrum pectinatum* (IC_50_ 67.55 mg/mL), the methanol extract of *Panus conchatus* (IC_50_ 72.83 mg/mL), and the methanol extract of *Aleuria aurantia* (IC_50_ 73.38 mg/mL) showed the weakest antioxidant activity. The results obtained are shown in Table 5.

### 3.5. Determination of Selected Minerals by ICP-OES

The highest amounts of calcium, magnesium, iron and zinc were determined in *Phallus hardiani*. The highest amounts of potassium, sodium, and phosphorus were determined in *Aleuria aurantia*; *Geastrum pectinatum* contained the most silicon, copper and manganese. The results are presented in Table 6.

### 3.6. Determination of Aluminum and Heavy Metals by ICP-OES

The highest contents of nickel, lead and aluminium were determined in *Geastrum pectinatum* (3.40 ppm, 0.714 ppm, and 83.3 ppm). In a *Panus conchatus* and *Aleuria aurantia* sample, lead and nickel were below the limit of quantification. The highest cadmium content was in *Aleuria aurantia* at 0.449 ppm. Cobalt in all samples was below the limit of quantification. The results are shown in Table 7.

### 3.7. Thermogravimetric Stability

The thermal stability of the analysed samples was determined based on the thermogravimetric results. All mushroom samples underwent a two-stage decomposition process. The first stage (at 51.2–57.4 °C) resulted from the decomposition of water adsorbed on the surface of the samples, while the second stage (at temperatures of 228.7–259.4 °C) caused weight loss. The temperature at the maximum rate of mass change was 301.7–319.7 °C, depending on the sample.

Of the samples analysed, the *Aleuria aurantia* sample was the most stable, showing stability up to 259 °C. At lower temperatures, samples of *Panus conchatus* (256 °C), and *Geastrum pectinatum* (246 °C) decayed. The sample *Phallus hadriani* (229 °C) decomposed at the lowest temperature. The results are summarised in Table 8.

## 4. Discussion

The broad applicability of the fungal is mainly due to the biodiversity of the chemical compounds they contain, which have diverse physiological effects. The most important bioactive components found in the mycelium or fruiting body include polysaccharides forming the cell wall structure (α- and β-glucans, peptidoglycans, heteroglycans, and polysaccharide-protein complexes), immunomodulatory proteins, lectins, terpenes, phenolic compounds, antioxidants with different mechanisms of action, laccase and fatty acids [6,7].

The four mushroom species analysed in our study (*Aleuria aurantia*, *Panus conchatus*, *Geastrum pectinatum*, and *Phallus hadriani*) contained different bioactive components with varying effects on human health. The highest amount of constituents were identified in *Panus conchatus* and *Phallus hadriani*.

Oleamide identified in *Panus conchatus* has been shown to have beneficial effects on the central nervous system [70]. Recent studies also suggest that oleamide has antidepressant, sleep-inducing [71], anticancer [72], and muscle atrophy-treating [73] properties. Nearly 60% of oleamide was also contained in *Phallus hadriani* and more than 20% in *Geastrum pectinatum*. The composition of *Panus conchatus* also contained decanoic acid, which, contrary to previous opinions, was found in a recent study not to be associated with the incidence of coronary heart disease [74,75]. There are also reports suggesting benefits from its consumption in the treatment of epilepsy, cancer, bipolar affective disorder and Alzheimer’s disease [76,77]. Diacylglycerols, of which 1,2-dipalmitoyl glycerol was the representative found in *Panus conchatus*, are absorbed and digested by a different metabolic pathway than conventional fats and oils. They show the ability to inhibit body fat accumulation, lower postprandial serum triacylglycerol, cholesterol and glucose levels and prevent osteoporosis [78]. Due to these properties, diacylglycerols are very interesting as a low-calorie fat substitute. In addition, their beneficial effect on product texture has led to their inclusion in various food matrices since 1999 [79,80]. *Panus conchatus* also contained small amounts (~4%) of linoleic acid (LA), which belongs to the essential fatty acids. However, the health effects of LA are currently controversial, given the significant increase in the amount of LA consumed in the standard diet. Excess LA has pro-inflammatory effects and leads to the formation of oxidised linoleic acid metabolites (OXLAM), impaired mitochondrial function and probably contributes to many chronic diseases [81,82].

*Geastrum pectinatum*, due to its hard and membranous endoperidium, is inedible. However, it can be used as a good source of 2-methylenocholestan-3-ol, belonging to the plant steroids (mycosterols), which, like other phytosterols, has proven efficacy in the prevention and treatment of cardiovascular diseases [83,84,85], immunomodulatory, regenerative, antidiabetic effects, and neurodegenerative diseases and impacts intestinal microflora and hormonal balance [86,87]. There are also suggestions for the possible transformation of plant sterols into sex steroids in mammals [87]. Some studies have shown that the hexadecanoamide found in *Geastrum pectinatum* in more than 8% has antioxidant, antiallergic, neuroprotective, and anti-inflammatory effects [88]. The steroid derivative iso-allocholate, found in 12%, has anti-inflammatory, antimicrobial, anti-asthmatic, and diuretic properties, as well as antiviral properties for SARS-CoV-2 [89,90].

*Aleuria aurantia* had an interesting composition with a high content of elaidic acid, an isomer of oleic acid with a trans configuration (over 77%). The effects of trans-configuration fatty acids (TFAs) on the body vary depending on their origin, composition, type and structure. Industrially produced TFAs have atherogenic effects, exacerbate insulin resistance, and increase the risk of breast and colorectal cancer, inflammation and body weight [91]. However, the health effects of natural TFAs, synthesised by bacterial metabolism of unsaturated fatty acids in ruminants, are still questionable [92,93]. Given that mushrooms contain trace amounts of fat, the adverse effects of TFAs may not be significant in this case. No less, the impact of elaidic acid derived from plant sources on the body seems to be an interesting material for exploration. *Aleuria aurantia* also contains palmitic acid, which belongs to the saturated fatty acids that have long been considered harmful, especially in cardiovascular disease. Recently, the negative effects of saturated fats on the human body, including the risk of cardiovascular disease, have been questioned [94,95]. Indeed, palmitic acid plays an important role in many pathological processes as a molecular signal transmitter [95]. However, it appears to be problematic only when it is synthesised as a result of diet-induced de novo lipogenesis (primarily as a result of sustained positive energy balance and excessive carbohydrate intake (particularly fructose) [95,96]. In addition, it may play a specific structural and functional role in fetal and infant life [94] and participate in the biosynthesis of palmitoyl ethanolamide (PEA), an endogenous compound with analgesic and anti-inflammatory effects [96]. It also shows potent antimicrobial activity against drug-resistant Staphylococcus epidermidis and Enterococcus faecalis [97]. *Aleuria aurantia* also contains oleic acid and polysaccharides, which have proven beneficial effects on human health.

*Phallus hadriani* contained oleamide in the highest amount (59%), in addition to 9% (2-phenyl-1,3-dioxolan-4-yl)-methyl ester of oleic acid, caproic acid with an unpleasant cheese odour and caustic properties [98]. However, in human colon cancer cell lines showing anticancer effects and a viability-reducing impact on colon cancer cells [99]. Small amounts of oleic acid and deoxyspergualin were also determined in *Phallus hadriani*, the 15-deoxyspergualin (DSG) form of which has shown immunosuppressive effects and has been proposed as a drug for the treatment of lupus nephritis [100] and in ANCA-positive vasculitis (antinuclear cytoplasmic antibodies; small vessel vasculitis with the presence of serum antibodies directed against the cytoplasmic antigen of neutrophils and monocytes) [101].

The bioactive compounds we identified in the mushroom species studied do not exhaust their phytopharmacological potential. Many bioactive compounds have not yet been identified, and perhaps the foundations for new solutions in producing supplements, nutraceuticals, and food enrichment are within these molecules [102].

Regardless of species, all analysed mushrooms also proved good sources of polyphenols—compounds with a broad spectrum of antioxidant, anti-inflammatory, and detoxifying activities. Of the samples analysed, *Phallus hadriani* extracts had the highest percentage of polyphenols content. Lower than the aqueous extract from Inonotus obliquus, a fungus with proven antioxidant, antiviral, anti-inflammatory, and antiparasitic properties [103] and higher than those in its methanol extract. The polyphenol content of the methanolic extract of Inonotus obliquus was comparable to *Aleuria aurantia* and *Panus conchatus* [104]. All the mushrooms we studied had a higher total polyphenol content than carrot, cabbage, cucumber, or fennel but lower than that of tomato, onion, lettuce and courgette. The percentage of polyphenols was higher in *Phallus hadriani* than in fruits except for pomegranate [105].

Overall, the tested samples showed significantly stronger antioxidant properties as determined with DPPH and ATBS compared to samples of arboreal fungi residing on Betula L [106]. However, they were weaker than those determined for vegetable and fruit extracts, except banana, which had weaker antioxidant properties than the aqueous extracts of *Phallus hadriani* [105]. They also showed less antioxidant activity compared to yellow sweet clover extract, white sweet clover herb, and yellow sweet clover herb commercial extract [107], having immunomodulatory and antioxidant, anti-edematous, and anti-inflammatory properties [108,109]. However, this does not diminish the possibility of their use as an ingredient in dietary supplements or functional foods, given that, of 43 selected Chinese mushrooms, shiitake mushrooms did not show the strongest antioxidant properties at all [110].

Mushrooms are a good source of minerals, including calcium, selenium, iron, manganese, zinc, and copper [111], as confirmed by our study. Significantly, by modifying the composition of the substrate, their mineral composition can be shaped [112,113]. As in other studies, we found varying mineral contents [114]. The phosphorus content of the tested fungal species varied within rather large limits; for *Aleuria aurantia*, it was higher, and for the other fungi, it was lower than that found for fungi such as Boletus edulis, Imleria badia, and Leccinum scabrum. Potassium was present in *Aleuria aurantia*, *Phallus hadriani*, and *Panus conchatus* in higher amounts than in Boletus edulis, Imleria badia, and Leccinum scabrum. *Phallus hadriani* was a much better source of magnesium, and the other mushrooms tested contained magnesium in amounts comparable to Boletus edulis, Imleria badia, and Leccinum scabrum [115]. *Phallus hadriani* and *Geastrum pectinatum* also contained more calcium than other mushrooms growing in Poland, Japan, Germany, or Finland. Only mushrooms from Turkey matched them in calcium content [115]. Fungi have a high capacity to accumulate heavy metals, and their concentrations sometimes exceed the amounts found in the substrate on which they grow. This phenomenon often occurs for cadmium compounds [116]. However, in our study, no exceedances of the permissible concentration of cadmium were observed in any of the fungi analysed. Lead content was also below acceptable levels [117]. In *Geastrum pectinatum*, the nickel content was too high concerning the revision of the EFSA (European Food Safety Authority) opinion considered at the request of the European Commission, which provides for a maximum level of 1 ppm for this component [118]. Aluminium content was also found to be relatively high, but in all analysed species, it was much lower than in dried tea, which is the richest dietary source of this nutrient [119].

The low thermal stability of the product limits its use in the food, catering or household industries. Meanwhile, the demonstrated high thermal stability, exceeding 250 °C for *Aleuria aurantia* and *Panus conchatus*, allows thermal and industrial processes to reach high processing temperatures without loss of nutritional and functional properties. All samples analysed had higher thermal stability than green coffee and its bioactive compounds (monochlorogenic and dichlorogenic acids and caffeine) [120]. Similar thermal stability was shown for turmeric, which is used as a supplement and included in functional foods, and slightly higher for cloves and cinnamon. *Geastrum pectinatum* and *Phallus hadriani* samples should be processed at lower temperatures, reaching 229 °C, similar to ginger, fenugreek, and garlic, which are stable in the temperature range of 170–220 °C [121].

## 5. Conclusions

Our study showed that all the mushrooms studied had a diverse content of bioactive compounds with a broad spectrum of health-promoting activities and could provide material for the extraction of compounds with physiological effects. *Panus conchatus* can provide a source for the extraction of compounds such as oleamide, decanoic acid, linoleic acid, and 1,2-dipalmitoylglycerol. Oleamide was also identified in *Phallus hadriani*; in addition, oleic acid, (2-phenyl-1,3-dioxolan-4-yl)-methyl ester of oleic acid, caproic acid, and deoxyspergualin were also present in this species. In addition to oleamide, *Geastrum pectinatum* can be a source of plant steroids such as 2-methylene-cholestan-3-ol, hexadecanoamide, and isoallochan a LF of 2,6-diaminopurine and adenine. *Aleuria aurantia* also has an interesting composition with a high content of elaidic acid, palmitic acid, and oleic acid.

In addition, the demonstrated presence of polyphenols and minerals, as well as antioxidant properties of varying potential and the absence of exceeding the permissible concentration of heavy metals, indicates that these species may be interesting material in the design of foods with health-promoting properties, nutraceuticals or dietary supplements.

The inclusion of the analysed mushrooms in the wide range of products offered by the food industry is also an innovative way to improve the thermal stability of the product, as confirmed by thermogravimetric studies. However, the use of the fruiting bodies of these mushrooms requires mandatory toxicological and clinical studies that meet the criteria of evidence-based medicine (EBM).

## Figures and Tables

**Table 1:** Characteristics of the study material.

Sample Symbol	Name	Date of Collection	Forest Type	Method of Drying the Fruiting Bodies	Suitability for Consumption
AA	*Aleuria aurantia*	October 2018	Mixed	Freeze-dried	Edible
PH	*Phallus hadriani*	September 2018	Leafy	Freeze-dried	Only the young fruiting body is edible; the mature fruiting body is inedible (gives off a faecal odour).
PC	*Panus conchatus*	October 2019	Mixed	Freeze-dried	Edible
GP	*Geastrum pectinatum*	September 2019	Coniferous	Dried at room temperature	Inedible

**Table 2:** Mineralisation parameters.

Power [W]	Rise Time [min]	Pressure [PSI]	Maximum Temperature [°C]	Temperature Holding Time [min]
600	20	195	210	10

**Table 3:** Percentage content of total polyphenols in fruiting bodies of analysed mushrooms.

Name	n	X ± SD [mg GAE/100 g s.m]	V% [%]
*Aleuria aurantia*	5	0.906 ± 0.025	2.8
*Phallus hadriani*	5	1.501 ± 0.025	1.7
*Panus conchatus*	5	0.900 ± 0.024	2.7
*Geastrum pectinatum*	5	0.617 ± 0.016	2.7

n—sample size, X—mean, SD—standard deviation, V%—coefficient of variation.

**Table 4:** IC_50_ (DPPH) parameter values for methanolic and aqueous extracts from the analysed fungi.

Name	n	IC_50_ ± SD [mg/mL]	V [%]
Methanolic extract			
*Aleuria aurantia*	5	40.86 ± 0.98	2.4
*Phallus hadriani*	5	18.19 ± 0.88	4.8
*Panus conchatus*	5	35.21 ± 1.30	3.7
*Geastrum pectinatum*	5	84.13 ± 2.10	2.5
Aqueous extracts			
*Aleuria aurantia*	5	17.06 ± 0.88	5.2
*Phallus hadriani*	5	4.74 ± 0.32	6.8
*Panus conchatus*	5	21.26 ± 1.24	5.8
*Geastrum pectinatum*	5	37.92 ± 2.60	6.9

IC_50_—IC50 (DPPH) parameter values, n—sample size, SD—standard deviation, V%—coefficient of variation.

**Table 5:** IC_50_ parameter (ABTS) values for methanolic and aqueous extracts from the samples analysed.

Name	n	IC_50_ ± SD [mg/mL]	V [%]
Methanolic extract			
*Aleuria aurantia*	5	73.38 ± 6.21	8.4
*Phallus hadriani*	5	31.71 ± 3.01	9.5
*Panus conchatus*	5	72.83 ± 5.44	7.5
*Geastrum pectinatum*	5	86.20 ± 4.12	4.8
Aqueous extracts			
*Aleuria aurantia*	5	53.43 ± 4.63	8.7
*Phallus hadriani*	5	10.21 ± 1.01	9.9
*Panus conchatus*	5	47.16 ± 2.06	4.4
*Geastrum pectinatum*	5	67.55 ± 5.21	7.7

IC_50_—IC_50_ parameter (ABTS) values, n—sample size, SD—standard deviation, V%—coefficient of variation.

**Table 6:** Content of selected minerals in the analysed mushroom samples.

Sample	Mineral Content [mg/g Dry Weight]				
	Calcium X ± SD	Copper X ± SD	Iron X ± SD	Sodium X ± SD	Magnesium X ± SD
*Aleuria aurantia*	0.238 ± 0.005	0.022± 0.001	0.067 ± 0.002	0.089 ± 0.002	0.988 ± 0.033
*Phallus hadriani*	8.977 ± 1.350	0.008 ± 0.000	0.14 ± 0.003	0.041 ± 0.001	3.402 ± 0.062
*Panus conchatus*	0.126 ± 0.002	0.005 ± 0.000	0.021 ± 0.002	0.066 ± 0.001	0.950 ± 0.011
*Geastrum pectinatum*	6.328 ± 0.170	0.037 ± 0.001	0.135 ± 0.012	0.040 ± 0.003	1.524 ± 0.040
	Zinc X ± SD	Silicon X ± SD	Potassium X ± SD	Phosphorus X ± SD	Manganese X ± SD
*Aleuria aurantia*	0.137 ± 0.002	<0	27.296 ± 0.311	13.970 ± 0.507	0.018 ± 0.000
*Phallus hadriani*	0.192 ± 0.002	0.064 ± 0.004	26.070 ± 0.843	4.687 ± 0.103	0.038 ± 0.000
*Panus conchatus*	0.021 ± 0.000	0.015 ± 0.002	11.350 ± 0.222	5.680 ± 0.218	0.015 ± 0.000
*Geastrum pectinatum*	0.087 ± 0.001	0.077 ± 0.003	18.900 ± 1.100	0.436 ± 0.025	0.133 ± 0.002

X—mean, SD—standard deviation.

**Table 7:** Content of heavy metal and aluminium in the analysed mushroom samples.

Heavy Metal and Aluminium Content [mg/g Dry Weight]					
Sample	Cadmium X ± SD	Lead X ± SD	Aluminum X ± SD	Nickel X ± SD	Cobalt X ± SD
*Aleuria aurantia*	0.0005 ± 0.0000	<0	0.0515 ± 0.0030	<0	<0
*Phallus hadriani*	0.0002 ± 0.0000	0.0005 ± 0.0002	0.1042 ± 0.0057	0.0007 ± 0.0001	
*Panus conchatus*	<0	<0	0.0075 ± 0.0003	<0	
*Geastrum pectinatum*	0.0003 ± 0.0000	0.0007 ± 0.0001	0.0833 ± 0.0048	0.0034 ± 0.0005	

X—mean, SD—standard deviation < 0—below the limit of quantification.

**Table 8:** Results of the thermogravimetric stability test of *Phallus hariani* (PH), *Panus conchatus* (PC), *Geatrum pectinatum* (GP), and *Aleuria aurantia* (AA).

Sample	Thermal Stability Temperature [°C]		Temperature at Maximum Rate of Mass Change [°C]		Weight Loss [%]	
	First Changes Stage 1	Layout Stage 2	First Changes Stage 1	Layout Stage 2	First Changes Stage 1	Layout Stage 2
PH	51.2 ± 1.03	228.7 ± 1.11	67.9 ± 2.13	319.7 ± 4.11	6.56 ± 2.02	67.91 ± 2.28
PC	51.7 ± 2.01	256.8 ± 2.73	68.2 ± 3.01	311.6 ± 3.04	6.75 ± 1.01	71.40 ± 4.08
GP	57.4 ± 3.19	246.3 ± 5.10	67.9 ± 4.15	319.7 ± 4.23	6.59 ± 2.12	67.88 ± 9.02
AA	51.1 ± 0.08	259.4 ± 1.27	68.8 ± 2.13	301.7 ± 3.11	4.93 ± 0.16	67.60 ± 0.03

First stage (Stage 1) decomposition resulting from the decomposition of water adsorbed on the surface of the samples, second stage (Stage 2) decomposition due to weight loss.

## Data Availability

The original contributions presented in the study are included in the article, further inquiries can be directed to the corresponding author.
